# Pre-transplant Sarcopenic Obesity Worsens the Survival After Liver Transplantation: A Meta-Analysis and a Systematic Review

**DOI:** 10.3389/fmed.2020.599434

**Published:** 2020-12-16

**Authors:** Péter Jenö Hegyi, Alexandra Soós, Péter Hegyi, Zsolt Szakács, Lilla Hanák, Szilárd Váncsa, Klementina Ocskay, Erika Pétervári, Márta Balaskó, Bálint Eröss, Gabriella Pár

**Affiliations:** ^1^Institute for Translational Medicine, Medical School, University of Pécs, Pécs, Hungary; ^2^Clinical Medicine Doctoral School, University of Szeged, Szeged, Hungary; ^3^Division of Gastroenterology, First Department of Medicine, Medical School, University of Pécs, Pécs, Hungary; ^4^Szentágothai Research Centre, University of Pécs, Pécs, Hungary

**Keywords:** non-alcoholic steatohepatitis, sarcopenic obesity, liver transplantation, body composition, non-alcoholic fatty liver disease

## Abstract

**Background:** The rising prevalence of cirrhotic cases related to non-alcoholic steatohepatitis has led to an increased number of cirrhotic patients with coexistence of obesity and muscle mass loss, known as sarcopenic obesity (SO). In patients undergoing liver transplantation (LT), the presence of SO may worsen prognosis, and increase morbidity and mortality.

**Objective:** We aimed to evaluate the effect of the presence of pre-transplant SO on the outcomes of LT.

**Methods:** A comprehensive search was performed in seven medical databases for studies comparing morbidity and mortality of patients with and without SO after LT. The primary outcome was overall mortality in the short- (1 year), intermediate- (3 years), and long- (5 years) term. We calculated pooled relative risks (RRs) with 95% confidence intervals (CIs). Heterogeneity was quantified with I^2^-statistics.

**Results:** Based on the analysis of 1,515 patients from three articles, SO increased overall mortality compared to non-SO at short-, intermediate-, and long-term follow-up (RR = 2.06, 95% CI: 1.28-3.33; RR = 1.67, 95% CI: 1.10-2.51; and RR = 2.08, 95% CI: 1.10-3.93, respectively) without significant between-study heterogeneity for the short- and intermediate- term (I^2^ = 0.0% for both) and considerable heterogeneity for long-term follow-up (I^2^ = 81.1%).

**Conclusion:** Pre-transplant SO proved to be a risk factor after LT and was associated with two times higher mortality at short- and long- term follow-up. Since SO worsens the prognosis of patients after LT, the inclusion of body composition assessment before LT may help to plan a more individualized nutritional treatment, physiotherapy, and postoperative care and may improve morbidity and mortality.

## Introduction

Obesity and metabolic syndrome, which can lead to non-alcoholic fatty liver disease (NAFLD) are becoming increasingly common medical problems in the Western world. Approximately 25% of adults with NAFLD will progress to inflammatory non-alcoholic steatohepatitis (NASH), which facilitates the progression of liver fibrosis to cirrhosis and end-stage liver disease and, therefore, liver transplantation (LT). NASH has become the second leading underlying cause of liver disease among adults on the LT waiting list in the United States (1–3), and it is expected to become the leading indication for LT by 2030 (4, 5). These patients often develop obesity and sarcopenia simultaneously, coined as sarcopenic obesity (SO) (6).

In western countries, overweight and obesity are now endemic (7). Although obesity is often seen in transplant recipients, there is a lack of accurate long-term data on the body composition of patients after the procedure. Obesity is considered to be among the most significant threats in healthcare today (8). More than 32% of the US population is considered to be obese, based on the body mass index (BMI) cut-off of 30 kg/m^2^ (9, 10). It is common knowledge that obesity increases the risk of perioperative complications (11) but how it affects the outcomes of LT in the long-term remains unclear. Studies have demonstrated that sarcopenia is an independent predictor of mortality, sepsis, and a more extended hospital stay after living donor LT (12–14). However, the exact mechanisms by which sarcopenia elicits poor prognosis are unclear (15). In the meta-analysis of van Vugt et al. (16), who discuss the association of skeletal muscle mass and the outcomes of LT in subjects from 19 studies (3,803 patients), sarcopenia was common with a prevalence ranging from 22 to 70%. The analysis revealed an inverse association of low muscle mass with post-LT mortality as well as a borderline inverse association with waiting list mortality [pooled hazard ratios (HRs) with 95% confidence intervals (CIs) were HR = 1.84, CI: 1.11-3.05 and HR = 1.72, CI: 0.99-3.00, respectively] (16). However, the authors did not analyze the impact of SO and assessed only overall survival.

Even though malnutrition and sarcopenia play an essential role in determining the prognosis of patients with liver cirrhosis (17), body composition analysis is frequently missing in clinical assessment, partly because it is often a clinical challenge to determine cirrhotic patients with fluid retention (18). Moreover, patients with NASH cirrhosis may develop a parallel loss of skeletal muscle and gain adipose tissue, which means that they develop SO (8). Sarcopenic muscle depletion is characterized by undesired changes on the body, such as reduced muscle size and an elevated intermuscular to intramuscular fat ratio, mitochondria dysfunctions, and systemic inflammation (19).

Based on the suggestions of other studies, this meta-analysis and systematic review explores whether SO is predictive of increased mortality in patients with cirrhosis (20, 21). The review focuses on current knowledge regarding the clinical impact of pre-transplant SO on post-transplant outcomes in LT.

## Materials and Methods

This meta-analysis and review are reported following the PRISMA Statement (2009) (22). This study is registered in PROSPERO priori under registration number CRD42019137574.

### Search

A systematic literature search was conducted by two independent reviewers (PH and ZS) for articles that discussed the effect of SO on outcomes of LT up to March 27, 2019. The search covered seven databases (MEDLINE via PubMed, EMBASE, Scopus, Web of Science, WHO Global Health Library, ClinicalTrials.gov, and CENTRAL) with the query **“*liver transplantation” AND*** (sarcopeni^*^OR sarcopaeni^*^ OR myopeni^*^ OR myopaeni^*^ ***OR “body composition” OR “lean body” OR “muscle mass” OR “muscle atrophy” OR*** **“*muscular atrophy” OR “muscle depletion” OR “core muscle” OR “muscle strength”) AND*** (obes^*^OR “fat mass” OR overweight^*^). No restrictions were imposed on the search.

### Inclusion and Exclusion Criteria

We used the ***PECOS***format to formulate our review question. We included studies which ***(P)***discussed adult patients after LT with different etiology [due to alcoholic liver disease, chronic viral hepatitis, NASH, and autoimmune hepatitis and also patients transplanted because of hepatocellular carcinoma (HCC)], and compared ***(E)***patients with radiologically-proven SO to ***(C)***those with non-SO body composition. We considered patients to have SO by adhering to the definitions of individual studies, all other patients were included in the non-SO group. Outcomes ***(O)***included peri- and post-transplant clinical outcomes. The primary outcome was overall mortality on short- (1 year), intermediate- (3 years), and long-term follow-up (5 years). Additional outcomes included operation time, perioperative blood loss, intraoperative erythrocyte transfusion requirement, and cold ischemic time. As regards the study design ***(S)***, we narrowed the focus to case-control studies and prospective and retrospective cohort studies (regardless of the publication type, i.e., abstract or full-text format). If there were multiple publications on the same cohorts of patients, the larger study population was included.

### Selection

Duplicates were removed with EndNote X7.4 (Clarivate Analytics, Philadelphia, PA, US), then title, abstract and full-text screening was performed by the two reviewers (PH and ZS) against the eligibility criteria. Disagreements were resolved by consensus.

### Data Collection

Data were independently extracted from studies and added to a pre-defined Excel datasheet (Office 365, Microsoft, Redmond, WA, US) by two reviewers in duplicate (PH and ZS). These included data on study setting (design, geographical region, centers, recruitment period), the essential characteristics of the study population (age, gender distribution, and etiology subtypes for LT), diagnostic criteria for SO, and outcomes with timing. We attempted to contact the corresponding authors of the relevant articles via email to obtain further data (20, 23, 24).

### Risk of Bias and Quality Assessment

We used Quality in Prognosis Studies (QUIPS) to assess the studies included as per the manual of use (25). Details of assessment are presented in Supplementary Appendix 1.

### Statistical Analysis

All meta-analytical calculations were performed by Stata 15.1 data analysis and statistical software (Stata Corp LLC, College Station, TX, USA) by a statistician (AS).

The available data allowed us to perform the analysis only on overall mortality. The reference group of comparison was the SO group. We calculated pooled risk ratios (RR) with CIs with the random-effects model using the Der Simonian–Laird method (26). The result of the meta-analysis was displayed graphically using forest plots. We performed separate analyses for short-, intermediate-, and long-term follow-up, as defined in the PECOS (see above).

Heterogeneity was tested by using Cochrane's Q and the *I*^2^ statistics, where *I*^2^ = 100% × (Q–df)/Q, and represent the magnitude of heterogeneity (moderate: 30–60%, substantial: 50–90%, considerable: 75–100%) (27).

If at least three studies were included in an analysis, we performed a sensitivity analysis by testing the effect of each study on the main association.

## Results

### Search and Selection

**Figure 1:**
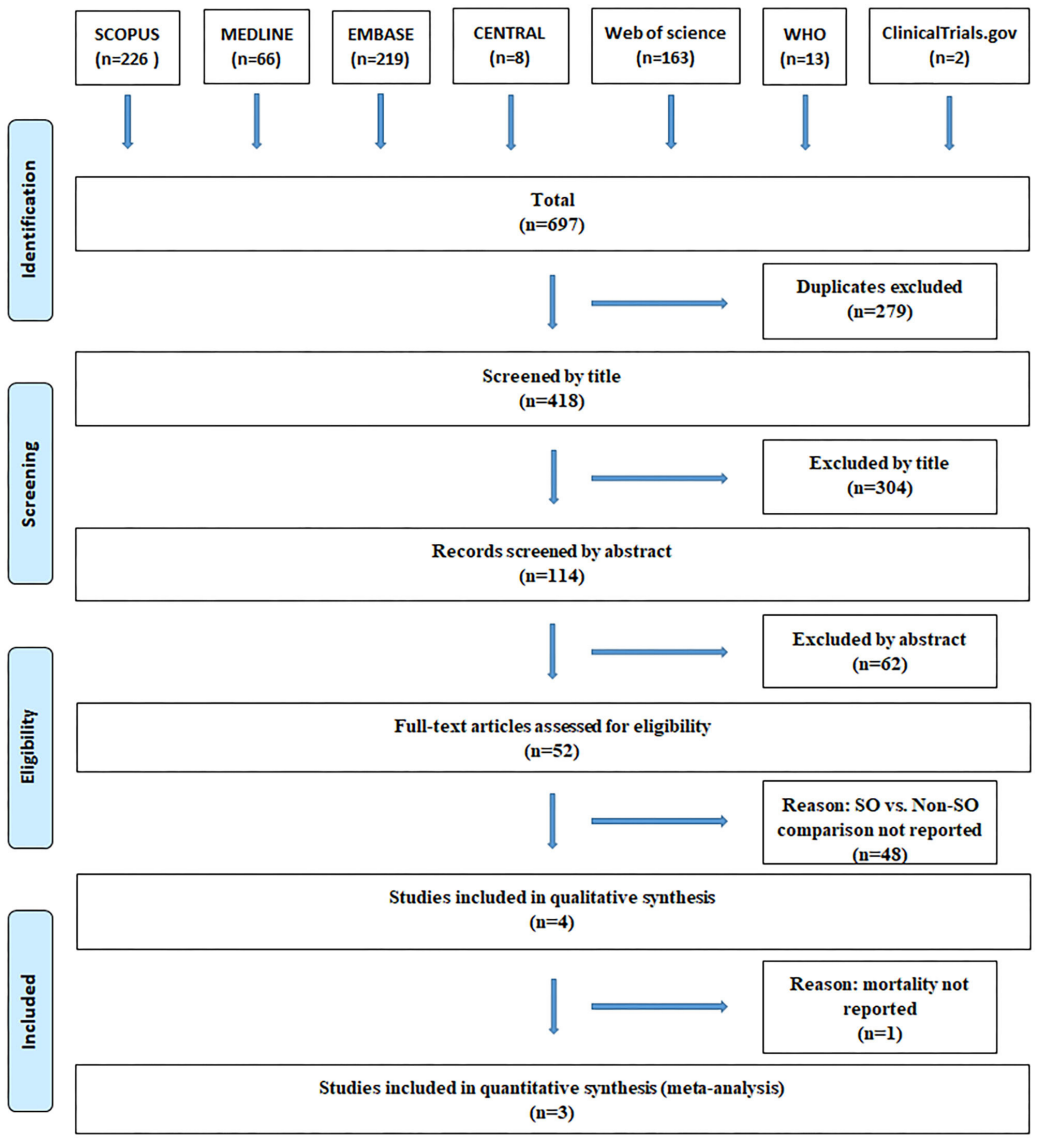
PRISMA flowchart.

The flow chart of the selection process is detailed in Figure 1. Using the search query, we identified 697 records in seven databases for evaluation, 66 in MEDLINE, 219 in Embase, 8 in CENTRAL, 226 in Scopus, 163 in Web of Science, 13 in WHO Global Health Library, and 2 in ClinicalTrials.gov. After the removal of duplicates and careful selection, 5 articles were judged to be eligible for inclusion (20, 21, 23, 24, 28), 2 of which discussed an overlapping study population (21, 24), from these, we kept the article including more patients (24) and excluded the other (21). Altogether, four papers were included in the systematic review, three of which were eligible for meta-analysis.

### Characteristics of the Studies Included

The main characteristics of the studies included are summarized in Table 1. Two articles recruited subjects from North-America (20, 28), another two from Japan (23, 24). All were retrospective cohort studies. By etiology of liver disease, 3 papers included mixed populations (20, 24, 28), while 1 included only patients with HCC (23).

**Table 1:** Characteristics of the studies included.

**References**	**Country (center)**	**Study type**	**Recruitment period**	**N^**0**^ of LT cases**	Etiology of the underlying liver disease^*^	**Sarcopenia**			**Obesity**		**Sarcopenic obesity**
						**Imaging techniques**	**Index**	**Cut-off**	**Index**	**Cut-off**	
Carias et al. (28)	The US (single center)	Retrospective cohort	2008–2013	207	Alcohol: 25% HCV: 23% NASH: 22% **HCC: 25%**	CT scan at the level of the L3 vertebra or DEXA	SMI	<38.5 cm^2^/m^2^ for females and <52.4 cm^2^/m^2^ for males	BMI	≥30 kg/m^2^	13%
Itoh et al. (23)	Japan (single center)	Retrospective cohort	2001–2012	153	HCV: 72% **HCC: 100%**	CT scan at the level of the L3 vertebra	SMI	Not reported	VFA	Not reported	33%
Kamo et al. (24)	Japan (single center)	Retrospective cohort	2008–2016	277	HCV: 33.6% NASH: 4% Biliary atresia: 20% Other: 31.4% **HCC: 27%**	CT scan at the level of the L3 vertebra	SMI	<40.31 cm^2^/m^2^ for males and <30.88 cm^2^/m^2^ for females	VFA and BMI	≥100 cm^2^ for VFA, ≥25 kg/m^2^ for BMI	25%
Montano-Loza et al. (20)	Canada (single center)	Retrospective cohort	2000–2013	678	Alcohol: 25% HCV: 43.3% HBV: 6.9% NASH: 15.5% AIH: 8.9% Other:0.8% **HCC: 43%**	CT scan at the level of the L3 vertebra	SMI	<41 cm^2^/m^2^ for females and <53 cm^2^/m^2^ for males	BMI	≥25 kg/m^2^	20%

* *The sum of etiologies exceeds 100% due to the overlap between the different causes of liver transplantation. Bold highlights indicate the proportion of HCC in the study population. AIH, autoimmune hepatitis; BMI, body mass index; CT, computed tomography; DEXA, dual-energy x-ray absorptiometry; HBV, hepatitis B virus; HCV, hepatitis C virus; HCC, hepatocellular carcinoma; LT, liver transplantation; NASH, non-alcoholic steatohepatitis; SMI, skeletal muscle mass index; VFA, visceral fat area*.

Body composition was assessed with CT scan in all studies approximately at the level of L3 vertebra, complemented with dual X-ray absorptiometry in 1 study (28). Sarcopenia was defined based on skeletal muscle mass index, while obesity was defined based on BMI and/or the visceral fat area in the articles. Since the cut-off values of the metrics differed across studies, SO was not uniformly defined in the individual articles, as detailed in Table 1.

### Findings of the Meta-Analysis and Systematic Review

**Figure 2:**
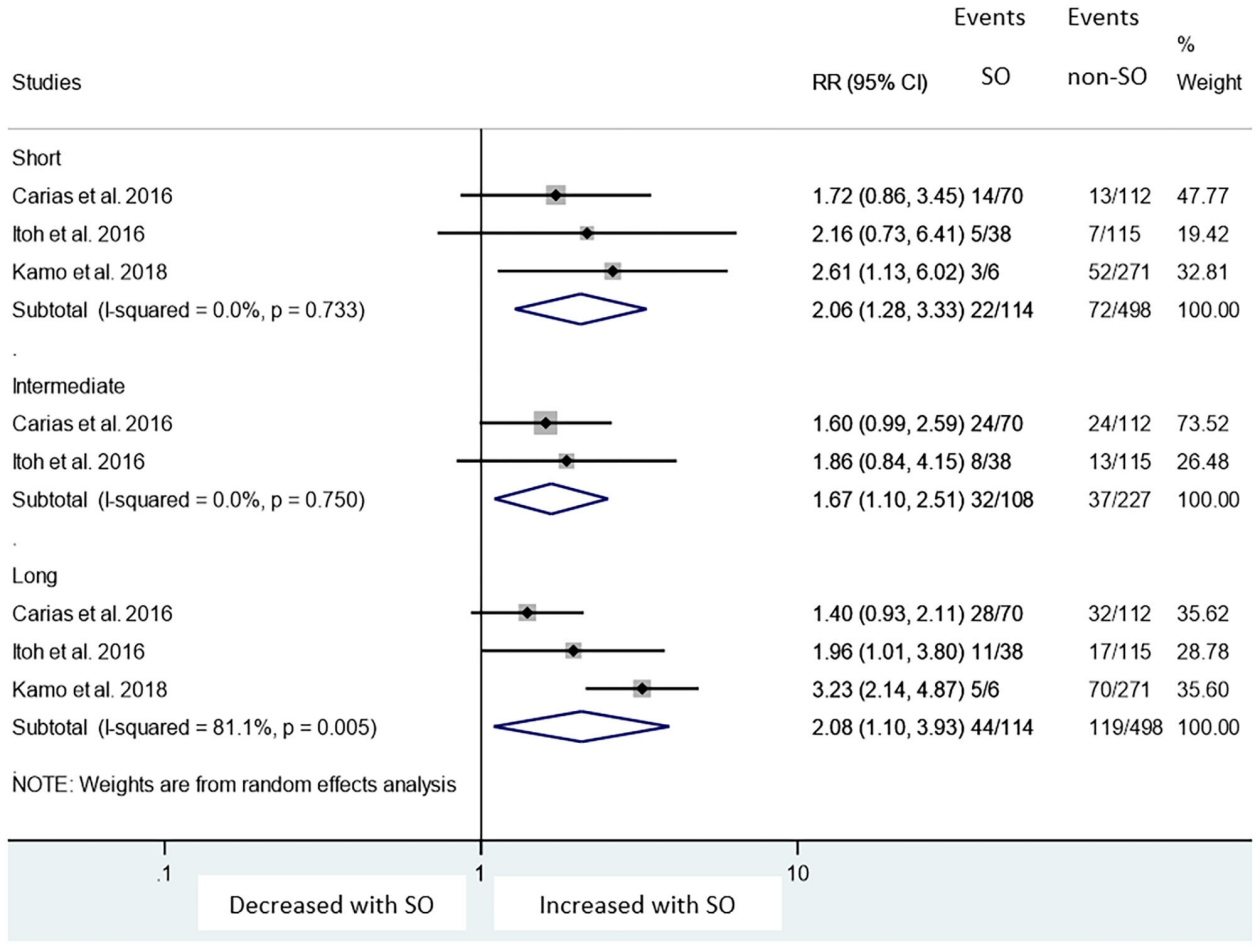
Risk ratios of mortality with sarcopenic obesity vs. non-sarcopenic obesity in short-, intermediate-, and long- term. Short-, intermediate-, and long- term follow-up mean 1, 3, and 5 years length of follow-up. RR, relative risk; CI, 95% confidence interval.

In our meta-analysis, we included 3, 2, and 3 articles to calculate mortality for short- (23, 24, 28), intermediate- (23, 28), and long-term follow-up (23, 24, 28), respectively. There were 114 vs. 498 patients in short-, 108 vs. 227 patients in intermediate-, and 114 vs. 498 patients in long-term follow-up in the SO and non-SO groups, respectively. SO significantly increased mortality on short-, intermediate-, and long-term follow-up (19 vs. 14%, RR = 2.06, CI: 1.28-3.33; 30 vs. 16%, RR = 1.67, CI: 1.10-2.51 and 39 vs. 24%, RR = 2.08, CI: 1.10-3.93, respectively) (Figure 2). When we omitted the studies one-by-one in sensitivity analysis, the direction of the main association changed only if we removed the study of Itoh et al. (23) from the analysis on long-term mortality (RR = 2.12, CI: 0.97-5.70), while [I^2^] reduced from 81.1 to 0.0%.

Three studies reported Kaplan-Meier curves for long-term follow-up (5 to 12 years). Although lower survival rates were reported for sarcopenic patients, there was no statistically significant difference between the groups in the analysis by Carias et al. (28). In the analysis provided by Kamo et al. (24), SO patients only had significantly worse survival compared to non-sarcopenic non-obese patients if the visceral fat area was used over the skeletal muscle index (*p* < 0.01 vs. *p* = 0.338) (24).

Patients transplanted for HCC with low skeletal muscle mass-to-visceral fat area ratio (SVR) had significantly worse overall and recurrence-free survival (*p* = 0.03 and *p* = 0.01) (23). In this study, SO proved to be an independent negative predictor of both recurrence-free survival and post-transplant mortality on a median follow-up of 5 years (HR = 5.26, CI: 2.03-13.8, *p* < 0.001 and HR = 2.58, CI: 1.17-5.52, *p* = 0.019, respectively).

One study reported that post-transplant mean survival (114 vs. 132 months, *p* = 0.1), length of hospital stay (35 vs. 31 days, *p* = 0.6), length of intensive care unit stay (8 vs. 8 days, *p* = 0.9), and the rate of bacterial infection (26 vs. 19%, *p* = 0.2) were statistically not different between SO and non-SO groups, respectively (20). In another study, perioperative mortality was higher in the SO group compared to non-SO patients (5.0 vs. 0.6%, respectively; *p*-value was not reported) (28).

### Risk of Bias and Quality Assessment of the Individual Studies

The summary of our risk of bias assessment is shown in Table 2. The adapted QUIPS tool and the details of the assessment can be found in Supplementary Appendix 1. The domain “study attrition” not fitting our meta-analysis were omitted due to the retrospective design of the included studies. Based on our analysis, the studies of Kamo et al. (24) and Carias et al. (28) were the highest-rated, with only one unclear domain of high risk of bias, while the studies of Itoh et al. (23) and Montano-Loza et al. (20) showed worse results, having two domains which carried high and another domain which carried an unclear risk of bias. Additionally, all the studies included were judged to be at high risk in one domain or more. The domain “study participation” had the highest-rate, as all the included studies were judged to be at low risk of bias. On the contrary, 100% of the studies were judged to be at high risk in terms of “study confounding,” since they failed to report how significant confounders were adjusted for and if an adequate method was used for treating missing data. “Prognostic factor measurement” and “outcome measurement” domains were assessed as having a low risk of bias in 75% of all studies. All studies carried an unclear risk of bias concerning “statistical analysis and reporting.”

**Table 2:** Quality of each included study.

**References**	**Study participation**	**Study attrition**	**Prognostic factor measurement**	**Outcome measurement**	**Study confounding**	**Statistical analysis and reporting**
Kamo et al. (24)	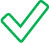	**n/a**	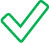	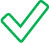	**X**	**?**
Montano-Loza et al. (20)	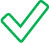	**n/a**	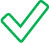	**X**	**X**	**?**
Itoh et al. (23)	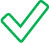	**n/a**	**X**	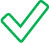	**X**	**?**
Carias et al. (28)	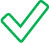	**n/a**	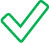	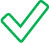	**X**	**?**

*Green ticks, red crosses, and yellow question marks represent a low, high, and unknown risk of bias, respectively. n/a: not applicable. Details of assessment are presented in Supplementary Appendix 1*.

## Discussion

### Summary of Findings

The effect of body composition changes, especially the wasting of skeletal muscles (sarcopenia) has been investigated and reported for various diseases (8).

Our meta-analysis is the first to examine the impact of SO, which is defined by the combination of low skeletal muscle mass index and either high visceral fat area or high BMI, on mortality after LT. The meta-analysis identified that pre-operative SO is associated with an almost two times higher mortality rate at short- intermediate- and long-term follow-up compared to non-SO groups (RR = 2.06, CI: 1.28-3.33; RR = 1.67, CI: 1.10-2.51; and RR = 2.08, CI: 1.10-3.93, respectively; Figure 2).

There are currently no generally accepted criteria for sarcopenia, which results in heterogeneous protocols and hinders efforts to generalize evidence. Measuring muscle strength remains easier and cheaper than measuring muscle mass. In the case of a more detailed and thorough clinical examination, the use of functional tests and more sophisticated methods (such as DEXA or computed tomography) should be considered (29).

Despite the rising prevalence of SO among LT patients, especially in the subgroup of NASH (11), the optimal management of obese and overweight LT candidates remained undetermined. Increased body mass index is commonly observed after LT (30), and it appears that much of this weight gain is an increase in fat mass (31). Although muscle function improves after organ transplantation within the first 3 months, the skeletal muscle remains below pre-transplant values (32). In an article by Carias et al. analyzing 207 patients, half of the patients were obese, 59% had sarcopenia, and SO was detected in 13% of them pre-transplant. Six months after transplantation sarcopenia was found in 95% of previously sarcopenic patients, of which 41.7% fulfilled the criteria for SO (28). Several studies identified independent pre-transplant predictors of mortality for post liver transplant patients. Kamo et al. (24) undertook a multivariate analysis and found that ABO incompatibility, low skeletal muscle mass index, high intramuscular adipose tissue content, and high visceral-to-subcutaneous adipose tissue area ratio, were independent risk factors of death. In a univariate analysis, BMI <25 kg/m^2^, graft type other than right graft, and operation time <12 h were also found to be risk factors. Itoh et al. (23) identified low muscle mass-to-visceral fat area ratio as an independent risk factor among cancer-specific variables. Chae et al. have found that more than 11.7% of the perioperative decrease of the psoas muscle index was also independently associated with survival after LT (33). Krell et al. found that pretransplant total bilirubin level is independently associated with a risk of developing severe infections and a worse 1-year survival rate (34). It is important to emphasize that appropriate post-transplant intervention, including nutritional therapy, rehabilitation with an evaluation of skeletal muscle mass and muscle functions, and physical activity interventions are recommended for better outcomes. Patients may benefit from inpatient rehabilitation programs that have been shown to decrease 30-day readmission rates (35). Although physical activity generally increases after LT, more than 75% of patients remain sedentary (36, 37). Regular exercise can optimize functioning after LT and should optimally begin in the pre-transplant setting (38). These justify the role of body composition measurements as part of the pre-transplant risk stratification and draw attention to the potential beneficial effect of the post-transplant correction of SO. The prognostic role of body composition was highlighted in HCC patients too. Based on the adjusted analysis of data on 1,257 patients, sarcopenia was an independent predictor of mortality (HR = 1.52; CI: 1.18-1.96) (39). Sarcopenia is also known to be associated with a higher complication rate, including hepatic encephalopathy, ascites formation, and infectious complications. SO is a risk factor not only for cirrhotic patients but also for those with cardiovascular diseases. Upadhya et al. confirmed the consistency between SO and the pathogenesis of exercise intolerance in heart failure with preserved ejection fraction in the elderly (40). Farmer et al. conducted a cohort study using the UK Biobank. They concluded associations between SO and the risk of cardiovascular disease and mortality. SO carried a high risk of developing heart failure, diastolic dysfunction, and impaired exercise capacity (41).

### Strengths and Limitations

This analysis is the first to assess mortality in sarcopenic obese patients who underwent liver transplantation. We distinguished between short-, intermediate-, and long-term mortality.

Although the original objective of this work was to analyze the effect of SO in patients who underwent LT due to NASH (as declared in the PROSPERO record), we were unable to perform the analysis due to lack of data. For the same reason, we could not compare patients with SO to those with normal and pathological body compositions (including sarcopenia and obesity alone). Instead, we performed an unplanned subgroup analysis on mortality for different time intervals.

The present meta-analysis involved data from only four articles. It must be noted, however, that we detected significant differences despite the limited study populations, excluding the chance of beta-type error. The number of studies prevented us from analyzing publication bias (<10 studies).

Since there is no consensus for the definition of SO (42), it was not uniform across studies nor was the way of body composition analysis (methodological heterogeneity, see in Table 1). This may also explain the divergence in the reported survival rates.

This study also has some limitations. The heterogeneity detected in the analysis of long-term survival may be explained by clinical heterogeneity due to the fact that the study of Itoh S et al. only included HCC population (23) (see, results of sensitivity analysis). On the other hand, there was no heterogeneity detected in the analysis of short- and intermediate term survival (homogenous datasets, see in Figure 2).

All the articles were published in North-America (20, 28) and Japan; (23, 24), meaning that data may not be representative of other geographical regions (6).

We do not have detailed information about the effects of covariates affecting survival (selection bias). However, the only study that adjusted the results for significant covariates, does agree with our results (23). Finally, all the included articles were retrospective cohort analysis, indicating a low level of evidence.

## Conclusion

### Implications for Practice

In conclusion, patients with SO showed worse survival after LT compared with non-SO patients. Abnormal body compositions including low skeletal muscle mass and visceral adiposity have substantial negative impacts on survival after LT, SO is associated with two times higher mortality both at short and long-term follow up. However, due to the coexistence of obesity with muscle mass depletion, sarcopenia might be overlooked. Since a CT scan is mandatory before LT, data are available on muscle mass, estimating body composition, and diagnosis of SO for all patients before LT. Clinicians should use this advantage by combining other simple-to-perform methods such as mid-arm muscle circumference measurements or assessment of skeletal muscle contractile function using a Handgrip strength method to detect malnutrition and take the available information into account when making a plan for the management of these patients both pre and post-transplant (43).

### Implications for Research

These results imply that the incorporation of body composition assessment into complex clinical prognostic scores (e.g., MELD or Child-Pugh score systems) may be beneficial, and should be tested under various clinical settings. Considering the global epidemics of obesity and type 2 diabetes, NASH is expected to become one of the leading causes of LT both for end-stage liver disease and hepatocellular carcinoma. Therefore, it is desirable to initiate further extensive prospective studies to explore the effect of all aspects of body composition, malnutrition, and SO on various outcomes of LT. Furthermore, the relationship between pre-transplant steroid therapy and the types of immunosuppressive treatment used after LT and SO should be investigated. Follow-up of body composition after transplantation (e.g., sarcopenia, obesity, and SO) should be undertaken to understand the complex effects of the pathophysiologic and therapeutic changes on sarcopenia after LT. Further research is needed to find out what factors affect the development of sarcopenic obesity after liver transplantation and what interventions could help reduce the high post-transplant mortality in patients with SO, using a standardized approach.

## Data Availability Statement

The original contributions presented in the study are included in the article/Supplementary Material, further inquiries can be directed to the corresponding author/s.

## Author Contributions

GP and PJH designed the research and study concept. PJH and ZS performed data extraction. AS analyzed and interpreted data. PJH and ZS performed quality and risk assessment. PJH, ZS, MB, EP, and BE wrote the article. KO and SV conducted the literature search and revised the manuscript. PH supervised the study. GP and PH conducted a critical revision of the manuscript for important intellectual content. All of the co-authors granted approved the version of the article for publication.

## Conflict of Interest

The authors declare that the research was conducted in the absence of any commercial or financial relationships that could be construed as a potential conflict of interest.
